# Clavicular non-union treated with fixation using locking compression plate without bone graft

**DOI:** 10.1186/s13018-018-1015-7

**Published:** 2018-12-13

**Authors:** Wan Chen, Kanglai Tang, Xu Tao, Chengsong Yuan, Binghua Zhou

**Affiliations:** Department of Orthopedic Surgery, Southwest Hospital, Third Military Medical University, Chongqing, 400038 China

**Keywords:** LCP, Clavicular non-union, Bone graft

## Abstract

**Background:**

The articles that have reported on the size at which a segmental defect of clavicular non-union requires bone grafting are scarce. This study evaluated the functional and radiologic results of fixation by locking compression plate (LCP) without bone graft when the defect size is less than 2 cm following bone sclerosis removal for the treatment of clavicular non-union.

**Methods:**

The study included 17 patients with mid-shaft clavicular non-union. All patients underwent bone sclerosis resection and fixation using LCP without bone graft. The patients were evaluated preoperatively, and after a minimum of 24 months (mean, 44.47 months; range, 24 to 60 months) postoperatively in terms of the disabilities of the arm, shoulder and hand (DASH) score, the Constant-Murley score, and radiography.

**Results:**

In this study, no patients were lost to follow-up. The mean DASH score improved from 38.76 ± 7.76 (31.00–46.52) points preoperatively to 19.88 ± 7.18 (12.70–27.06) points 2 years postoperatively (*P* < 0.01). The mean Constant-Murley score improved from 41.59 ± 8.81 (32.78–50.40) points preoperatively to 75.47 ± 13.50 (61.97–88.97) points 2 years postoperatively (*P* < 0.01). Radiographs revealed fracture union in all patients. No correlations between the defect size and the postoperative Constant-Murley score or between the defect size and the postoperative DASH score were found based on Pearson tests. No complications, particularly acromioclavicular joint complications and sternoclavicular joint complications, were observed.

**Conclusions:**

In conclusion, we can suggest, from the findings of our study, that bone sclerosis resection and fixation using LCP without bone graft is effective for the treatment of clavicular non-union involving a gap of less than 2 cm and has a low rate of complications.

## Introduction

Clavicle fractures account for up to 5 to 10% of all fractures [1]. Clavicular non-union is a rare complication and has been reported in between 0.1 and 24% of patients following non-operative treatment [2–5]. It can be disabling and presents mainly with pain and limitations of shoulder movement [6–8]. Contributing factors to clavicular non-union include clavicle shortening > 15–20 mm, female sex, fracture comminution, fracture displacement, older age, severe initial trauma, and unstable lateral fractures (Neer type II) [8]. Schnetzke et al. studied 58 patients with variations in the included types of clavicle fracture healing complications and reported rates of 33% atrophic, 20% hypertrophic, 7% mixed type, and 40% delayed fracture healing complications [9].

Symptomatic non-unions including functional shoulder impairment and pain are usually treated surgically. Surgical methods include resection of part of the clavicle or the entire clavicle and clavicle reconstruction. With the development of implant technology for anatomic reconstruction, clavicle resection has been abandoned. Currently, the general principles for the treatment of clavicular non-unions are the same as those for other fracture sites, i.e., bone union is achieved with fracture site reduction, stable fixation, and bone grafting transplantation from the site itself or the iliac crest [10, 11]. Fixation is often internal and must provide stability. Many authors recommend the use of bone grafts [2, 9, 10, 12]. The disadvantages of bone graft include the limited volume of available bone [13], increased operative time and blood loss [14], and donor-site morbidity [15, 16]. The articles that have reported on the size at which a segmental defect of the clavicular non-union requires bone grafting are scarce.

In recent years, we have treated non-unions of the clavicle with defects less than 2 cm (after bone sclerosis resection) directly using compressive locked-plate internal fixation without bone graft. Based on the evaluation of postoperative symptom relief, functional improvement of the shoulder, clavicle healing, and complications, we hypothesized that direct internal fixation using a LCP without bone graft for the treatment of clavicular non-unions would result in reduced pain levels, improved function of the shoulder, and promoted clavicular non-union but without complications.

## Materials and methods

A prospective clinical study was designed. The inclusion criteria for the patients consisted of the following: clavicular non-union lasting more than 6 months, functional shoulder impairment and pain, and defect after bone sclerosis removal of less than 2 cm. The exclusion criteria were the following: clavicular non-union lasting less than 6 months, no functional shoulder impairment and pain, and defect after bone sclerosis removal of more than 2 cm, infected non-unions, tumors, or pathologic fractures, patients with cancer or compromised immune systems, patients refusing surgical treatment. Between January 2009 and June 2014, 17 patients (12 men and 5 women; age range, 13 to 74 years; mean age, 38.5 years; 9 left side and 8 right side; 12 initially treated with non-operative treatment and 5 treated with fracture reduction and internal fixation) with clavicular non-union were included in this study. On standard A-P X-ray of the clavicle, 8 of the non-unions were atrophic (no osteogenesis) and 9 were hypertrophic. Non-union and non-union type was defined by the lack of both periosteal and endosteal healing responses and bridging of the fracture after 6 months. In cases of doubt, a CT scan was performed to support or reject the diagnosis based on conventional radiographic images and clinical signs, such as pain and weakness. The demographic data, injury-surgery time, clavicular non-union type, defect size of each patient, and initial treatment were recorded (Table 1). All patients reported shoulder pain and impairments of shoulder function. The preoperative radiographs revealed no other injuries. The operations and clinical follow-ups were conducted by two senior surgeons (K.T., X.T.). The defect sizes were measured after bone sclerosis resection.

**Table 1:** Patient details

Case no.	Age, years	Sex	Side	Time injury-surgery, months	Type	Defect size, cm	Initial treatment
1	52	F	R	9	H	1.1	C
2	45	M	R	18	H	0.9	S
3	37	M	R	17	H	1.6	C
4	44	F	R	11	H	2.0	C
5	42	M	L	34	H	1.4	S
6	59	F	L	25	A	1.1	C
7	31	M	L	52	A	0.6	C
8	13	M	R	7	H	0.7	C
9	27	F	L	11	H	0.8	S
10	47	M	L	10	A	1.4	C
11	16	M	R	28	H	1.4	C
12	41	M	L	18	A	1.6	S
13	26	M	R	21	A	1.3	C
14	74	M	L	35	A	0.9	S
15	31	F	R	7	A	1.3	C
16	27	M	L	27	A	1.6	C
17	42	M	L	7	H	1.0	C

*F* female, *M* male, *L* left, *R* right, *A* atrophy, *H* hypertrophy, *C* conservative, *S* surgery

### Clinical evaluation

The disabilities of the arm, shoulder and hand (DASH) scores and the Constant-Murley scores were used to evaluate the symptoms and functional level of each patient before and a minimum of 24 months after surgery. All patients underwent an X-ray before and after a minimum of 24 months to observe fracture union after surgery.

### Surgical technique

Under general anesthesia, the patient was placed in the supine position with a large bump placed between the scapula. A 6-cm incision was made along the clavicle anterosuperiorly, centered over the fracture site. A dissection was carefully performed to avoid dissecting the periosteum and any injury to the subclavian vessels or the anterior fibers of the brachial plexus, and the fracture position was fully exposed. An oscillating saw was used to remove bone sclerosis and/or the fracture callus until fracture end bleeding was observed and the fragments were smooth. The medullary canal was opened via Kirschner wire perforation. A steel ruler was used to measure the gap size. If the defect size was within 2 cm, the fragments were aligned and immobilized with a pointed reduction clamp and fixed with a LCP (Synthes, Switzerland). The LCP was placed on the anterosuperior side of the clavicle. The anatomic reduction and screw lengths were confirmed by fluoroscopy. If the defect was greater than 2 cm, bone grafting was used, and the patient was excluded from these analyses. The wounds were closed in a routine manner, and sterile compression dressings were applied.

### Postoperative rehabilitation

The operated extremity was placed in a sling for comfort. Pendulum (also known as Codman) exercises were taught to the patient, and the patient was encouraged to use the arm but to avoid heavy lifting, pushing, or pulling. Full return to activities was allowed when fracture healing was present, which was typically at 2 to 3 months.

### Statistics

The statistical analyses were performed using SPSS software version 13.0 (IBM, Armonk, NY). Paired-sample *t* tests and Mann-Whitney *U* tests were used to compare the DASH scores and Constant-Murley scores, respectively, before and after the procedures. Pearson tests were used to analyze the correlation between the defect size and the postoperative Constant-Murley scores or between the defect size and the postoperative DASH scores. The level of significance was set at 95%, and *P* < 0.05 was considered significant.

## Results

**Fig. 1:**
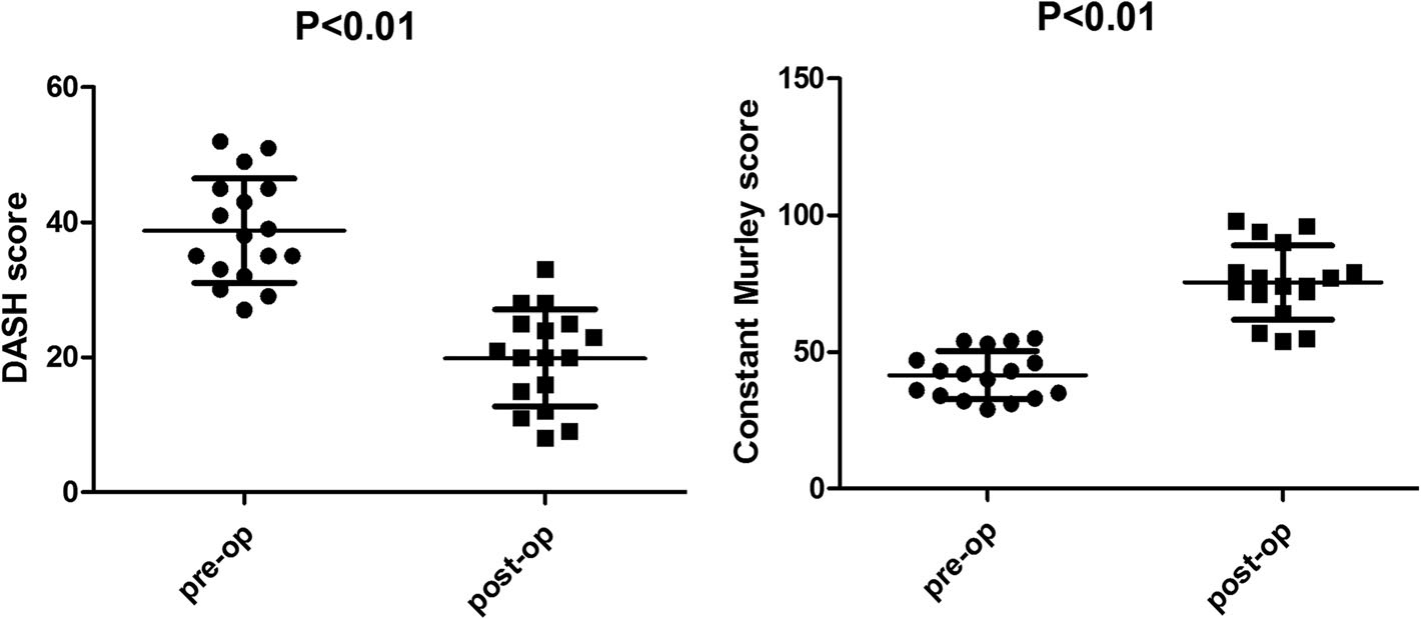
After the operations, the DASH scores significantly decreased (*P* < 0.01) in all 17 patients, and the Constant-Murley scores significantly increased (*P* < 0.01) in all patients

**Fig. 2:**
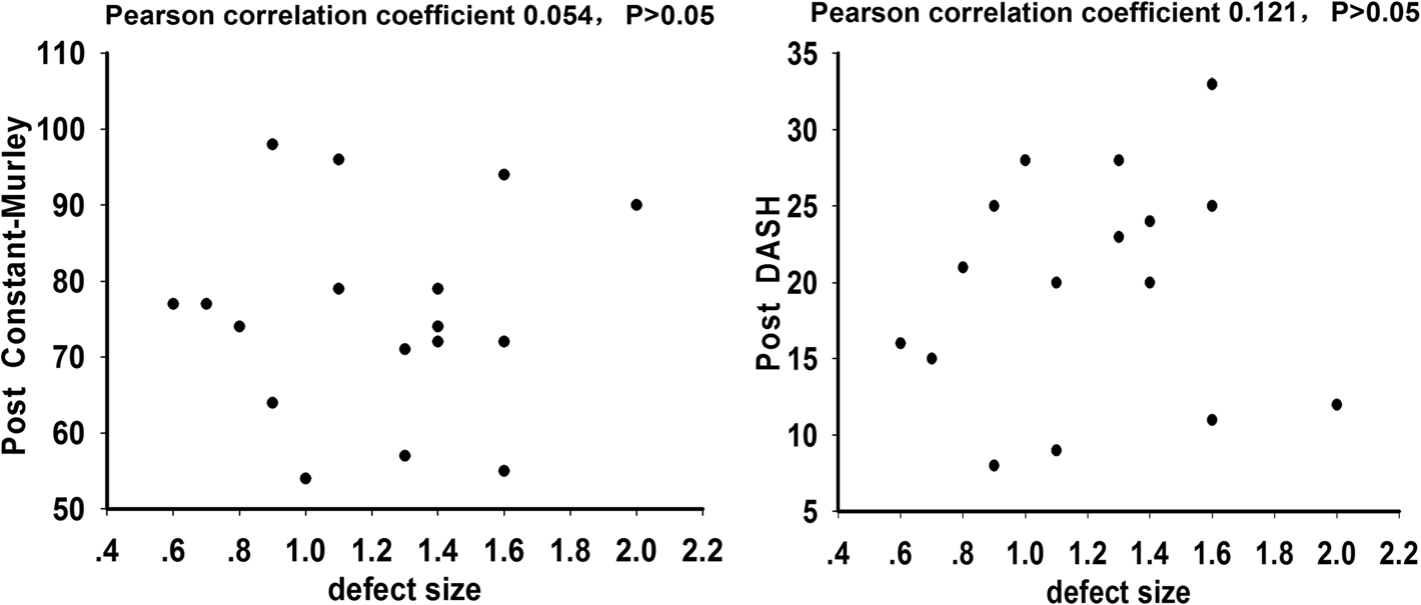
The Pearson correlation coefficients between defect size and the postoperative Constant-Murley score was 0.054, and the Pearson correlation coefficient between the defect size and postoperative Constant-Murley score was 0.121. Defect size was not correlated with the postoperative Constant-Murley score or the postoperative DASH score (*P* > 0.05)

**Fig. 3:**
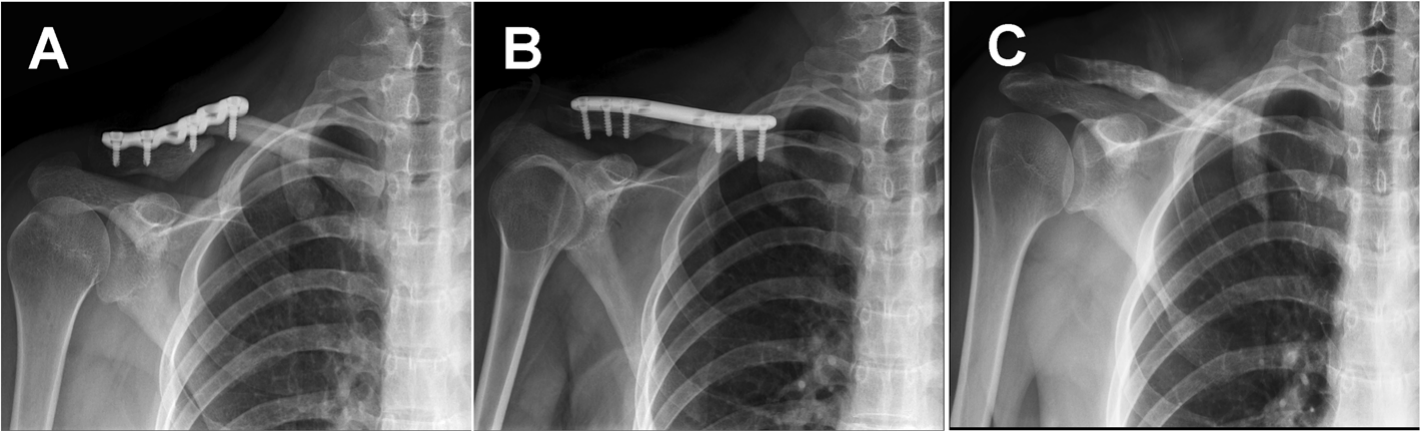
**a** Pre-operative radiograph of a clavicle fracture non-union (atrophic) in a 31-year-old female. **b** Immediate post-operative radiograph showing fixation with three screws on either side of the fracture. **c** One year after surgery, the internal fixation devices were removed. The final follow-up radiograph showing fracture union

According to the previously described criteria, 17 patients were included and treated with fixation using a LCP without bone graft; 3 patients were excluded due to that the defect size was more than 2 cm. The fracture sites of 9 patients were hypertrophy and 8 were atrophy. The mean follow-up time was 44.47 ± 14.55 (24–60) months. No patients were lost to follow-up. The mean DASH score improved from 38.76 ± 7.76 (31.00–46.52) points preoperatively to 19.88 ± 7.18 (12.70–27.06) points 2 years postoperatively (*P* < 0.01). The mean Constant-Murley score improved from 41.59 ± 8.81 (32.78–50.40) points preoperatively to 75.47 ± 13.50 (61.97–88.97) points 2 years postoperatively (*P* < 0.01) (Table 2, Fig. 1). No correlations between the defect size and the postoperative Constant-Murley score or between the defect size and the postoperative DASH score were found based on Pearson tests (Fig. 2). The radiographs revealed fracture union in all patients in the study group (Fig. 3). No complications, particularly acromioclavicular joint complications and sternoclavicular joint complications, were observed.

**Table 2:** Clinical evaluations of the patients

Case no.	DASH score		Constant-Murley score		Union or not	Follow-up time	Complications
	Pre-op	Post-op	Pre-op	Post-op			
1	27	9	55	96	Union	24	None
2	29	8	54	98	Union	48	None
3	30	11	54	94	Union	24	None
4	32	12	53	90	Union	60	None
5	33	20	47	79	Union	60	None
6	35	20	46	79	Union	24	None
7	35	16	43	77	Union	60	None
8	35	15	43	77	Union	36	None
9	39	21	40	74	Union	60	None
10	38	20	42	74	Union	48	None
11	41	24	36	72	Union	24	None
12	43	25	35	72	Union	36	None
13	45	23	33	71	Union	48	None
14	45	25	34	64	Union	60	None
15	49	28	32	57	Union	48	None
16	51	33	31	55	Union	36	None
17	52	28	29	54	Union	60	None
Mean	38.76 ± 7.76	19.88 ± 7.18	41.59 ± 8.81	75.47 ± 13.50		44.47 ± 14.55

## Discussion

There is a general agreement that fracture site reduction and stable fixation is a key procedure for promoting clavicle union. But there is no agreement in published studies on the requirement for bone substitutes. Most authors recommended bone grafting [2, 10, 17–19]. One study reported union rates of 71% with the use of bone graft in a series of 21 clavicular non-unions treated after the first surgery. Six complications required a revision procedure: three for infection and three for mechanical failure [10]. A retrospective study conducted by Schnetzke et al. revealed that bone graft transplantation can result in a significantly shorter time to bone consolidation, better clinical results in terms of the DASH and Constant-Murley scores, and lower revision rates compared with non-bone-graft transplantation [9]. However, it did not provide the defect size after preparing of grafting group and no grafting group. Harvesting of bone graft in itself can have a high morbidity rate, up to 30% in one study [20]. This includes graft site infection, pain, and fracture.

By contrast, others suggest that bone grafting may be unnecessary in every case of clavicular non-union. Some suggested bone grafts should be used only with atrophic non-unions [21–23]. Baker et al. reported on a series of patients with clavicular non-unions that were fixed with a pre-contoured locking plate and no bone graft. All of these patients returned to work and regular sports activities, and this finding is consistent with our series [21]. However, it did not provide information regarding the defect lengths of all patients after bone sclerosis removal, and it did not analyze the extent of clavicle shorting, which did not affect clinical outcomes. In the present study, the defect size of 17/20 was less than 2 cm, all 17 patients achieved pain relief and functional improvement, and the X-rays revealed clavicle fracture healing in all patients. No correlations between the defect size and the postoperative Constant-Murley score or between the defect size and the postoperative DASH score were found.

Matsumura et al. suggested in a biomechanical study that a clavicle shortening of 10% or more alters the scapula. Moreover, this author reported that the mean length of the clavicle was 158.1 ± 7.0 (151.1–165.1) mm (range, 144.0–176.4 mm) [24]. However, in the present study, a cutoff point was set for the defect of 2 cm, which slightly exceeds the 10% of the mean length of the clavicle reported by Matsumura, and clavicle shorting below 2 cm did not affect shoulder function. Maybe it is because Matsumura’s results were based on a cadaveric study.

Currently, there is no consensus regarding the optimal fixation devices for clavicular non-union. In a comparison of dynamic compression plating (DCP) in 16 patients and low-contact dynamic compression plating (LC-DCP) techniques in 17 patients, Kabak et al. reported that the use of LC-DCP is a more reliable treatment method than the use of the DCP because the LC-DCP has several technical advantages that make it an ideal implant for satisfying the unique anatomic and biomechanical requirements of the internal fixation of clavicular non-union [25]. In the present study, LCP was used to fix clavicular non-unions and obtained good outcomes. The LCP offers a low-profile solution for the plating of the clavicle. The titanium plate offers strength, with a rounded profile and a low-profile screw-plate interface, which is known to promote early callus formation [23].

There are several limitations to our study. First, the sample size was relatively small. The calculation of accurate cutoff point of defect size which helps to decide whether or not to transplant bone graft needs a bigger size sample and to conduct ROC curve analysis. Second, the follow-up time was relatively short. Third, there is no control group. Thus, additional high-level evidence like RCT is needed.

## Conclusions

In conclusion, we can suggest, from the findings of our study, that bone sclerosis resection and fixation using LCP without bone graft is effective for the treatment of clavicular non-union involving a gap of less than 2 cm and has a low rate of complications.

## Abbreviations

DASH: Disabilities of the arm, shoulder and hand
DCP: Dynamic compression plating
LC-DCP: Low-contact dynamic compression plating
LCP: Locking compression plate

## References

[1] Ramoutar DN, Rodrigues J, Quah C, Boulton C, Moran CG (2011). Judet decortication and compression plate fixation of long bone non-union: Is bone graft necessary?. Injury.

[2] Stufkens SA, Kloen P (2010). Treatment of midshaft clavicular delayed and non-unions with anteroinferior locking compression plating. Archives of Orthopaedic and Trauma Surgery.

[3] Nowak J, Mallmin H, Larsson S (2000). The aetiology and epidemiology of clavicular fractures. A prospective study during a two-year period in Uppsala, Sweden. Injury.

[4] Zlowodzki M, Zelle BA, Cole PA, Jeray K, McKee MD (2005). Evidence-Based Orthopaedic Trauma Working G. Treatment of acute midshaft clavicle fractures: systematic review of 2144 fractures: on behalf of the Evidence-Based Orthopaedic Trauma Working Group. Journal of orthopaedic trauma.

[5] Virtanen KJ, Remes V, Pajarinen J, Savolainen V, Bjorkenheim JM, Paavola M. Sling compared with plate osteosynthesis for treatment of displaced midshaft clavicular fractures: a randomized clinical trial. The Journal of bone and joint surgery American volume. 2012;94(17):1546-53. 10.2106/JBJS.J.01999 22832887

[6] George DM, McKay BP, Jaarsma RL. The long-term outcome of displaced mid-third clavicle fractures on scapular and shoulder function: variations between immediate surgery, delayed surgery, and nonsurgical management. Journal of Shoulder and Elbow Surgery. 2015;24(5):669-76. 10.1016/j.jse.2014.09.037 25457191

[7] van der Meijden OA, Gaskill TR, Millett PJ. Treatment of clavicle fractures: current concepts review. Journal of shoulder and elbow surgery / American Shoulder and Elbow Surgeons [et al]. 2012;21(3):423-9. 10.1016/j.jse.2011.08.053 22063756

[8] Martetschlaeger F, Gaskill TR, Millett PJ. Management of clavicle nonunion and malunion. Journal of Shoulder and Elbow Surgery. 2013;22(6):862-8. 10.1016/j.jse.2013.01.022 23562292

[9] Schnetzke M, Morbitzer C, Aytac S, et al. Additional bone graft accelerates healing of clavicle non-unions and improves long-term results after 8.9 years: a retrospective study. Journal of Orthopaedic Surgery and Research. 2015;10. 10.1186/s13018-014-0143-y PMC4296679 25573541

[10] Faraud A, Bonnevialle N, Allavena C, Degorce HN, Bonnevialle P, Mansat P. Outcomes from surgical treatment of middle-third clavicle fractures non-union in adults: A series of 21 cases. Orthopaedics & Traumatology-Surgery & Research. 2014;100(2):171-6. 10.1016/j.otsr.2013.09.011 24534201

[11] Riggenbach MD, Jones GL, Bishop JY (2011). Open reduction and internal fixation of clavicular nonunions with allograft bone substitute. International Journal of Shoulder Surgery.

[12] Myeroff C, Archdeacon M. Autogenous Bone Graft: Donor Sites and Techniques. Journal of Bone and Joint Surgery-American Volume. 2011;93A(23):2227-36. 10.2106/JBJS.J.01513 22159859

[13] Jones AL, Bucholz RW, Bosse MJ (2006). Recombinant human BMP-2 and allograft compared with autogenous bone graft for reconstruction of diaphyseal tibial fractures with cortical defects. A randomized, controlled trial. The Journal of bone and joint surgery American volume.

[14] Arrington ED, Smith WJ, Chambers HG, Bucknell AL, Davino NA (1996). Complications of iliac crest bone graft harvesting. Clinical orthopaedics and related research..

[15] Robertson PA, Wray AC (2001). Natural history of posterior iliac crest bone graft donation for spinal surgery: a prospective analysis of morbidity. Spine.

[16] Sasso RC, LeHuec JC, Shaffrey C. Iliac crest bone graft donor site pain after anterior lumbar interbody fusion: a prospective patient satisfaction outcome assessment. Journal of spinal disorders & techniques. 2005;18 Suppl:S77-81. 10.1097/01.bsd.0000112045.36255.83 15699810

[17] O'Connor D, Kutty S, McCabe JP (2004). Long-term functional outcome assessment of plate fixation and autogenous bone grafting for clavicular non-union. Injury.

[18] Olsen BS, Vaesel MT, Sojbjerg JO. Treatment of midshaft clavicular nonunion with plate fixation and autologous bone grafting. Journal of shoulder and elbow surgery / American Shoulder and Elbow Surgeons [et al]. 1995;4(5):337-44. 10.1016/s1058-2746(95)80017-4 8548436

[19] Fuchs B, Steinmann SP, Bishop AT. Free vascularized corticoperiosteal bone graft for the treatment of persistent nonunion of the clavicle. Journal of shoulder and elbow surgery / American Shoulder and Elbow Surgeons [et al]. 2005;14(3):264-8. 10.1016/j.jse.2004.06.007 15889024

[20] Younger EM, Chapman MW (1989). Morbidity at bone graft donor sites. Journal of orthopaedic trauma.

[21] Baker JF, Mullett H (2010). Clavicle non-union: autologous bone graft is not a necessary augment to internal fixation. Acta Orthop Belg.

[22] Endrizzi DP, White RR, Babikian GM, Old AB (2008). Nonunion of the clavicle treated with plate fixation: A review of forty-seven consecutive cases. Journal of Shoulder and Elbow Surgery.

[23] Khan SA, Shamshery P, Gupta V, Trikha V, Varshney MK, Kumar A (2008). Locking compression plate in long standing clavicular nonunions with poor bone stock. The Journal of trauma.

[24] Matsumura N, Ikegami H, Nakamichi N (2010). Effect of shortening deformity of the clavicle on scapular kinematics: a cadaveric study. The American journal of sports medicine.

[25] Kabak S, Halici M, Tuncel M, Avsarogullari L, Karaoglu S (2004). Treatment of midclavicular nonunion: Comparison of dynamic compression plating and low-contact dynamic compression plating techniques. Journal of Shoulder and Elbow Surgery.

